# Lung Adenocarcinoma Patient Harboring *EGFR*-KDD Achieve Durable Response to Afatinib: A Case Report and Literature Review

**DOI:** 10.3389/fonc.2021.605853

**Published:** 2021-04-07

**Authors:** Lingling Zhao, Zhen Wang, Haiwei Du, Songan Chen, Pingli Wang

**Affiliations:** ^1^Department of Respiratory and Critical Care Medicine, The Second Affiliated Hospital of Zhejiang University School of Medicine, Hangzhou, China; ^2^Burning Rock Biotech, Guangzhou, China

**Keywords:** afatinib, EGFR-KDD, lung adenocarcinoma, next-generation sequencing, tyrosine kinase inhibitor

## Abstract

The rapid development of epidermal growth factor receptor (EGFR)-tyrosine kinase inhibitors (TKIs) has revolutionized the treatment of patients with advanced or metastatic non-small cell lung cancer (NSCLC) harboring *EGFR* mutations including but not limited to exon 19 deletions (19 del) and point mutation L858R in exon 21. However, the efficacy of EGFR-TKIs in patients with rare mutations, such as *EGFR-*kinase domain duplication (KDD), remains elusive. *EGFR*-KDD often results from in-frame tandem duplication of *EGFR* exons 18–25, causing subsequent constitutive activation of EGFR signaling. Several case reports have revealed the efficacies of EGFR-TKIs in advanced lung adenocarcinoma (LUAD) with *EGFR*-KDD but yielded variable antitumor responses. In the present study, we report a 61-year-old male patient diagnosed with T1N3M0 (stage IIIB) LUAD harboring *EGFR*-KDD involving exons 18–25. He was treated with afatinib and achieved partial response (PR) with progression-free survival (PFS) of 12 months and counting. Our work, confirming *EGFR*-KDD as an oncogenic driver and therapeutic target, provides clinical evidence to administer EGFR-TKIs in patients with advanced LUAD harboring *EGFR*-KDD.

## Introduction

Lung cancer as the leading cause of cancer-related mortality worldwide accounts for almost one-quarter of all cancers (1). Non-small cell lung cancer (NSCLC) accounts for 85% of all lung cancer cases, including two major histological subtypes, adenocarcinoma and squamous cell carcinoma (2). With the advancements of sequencing technologies, it has been well-known that the initiation and development of some NSCLCs, especially lung adenocarcinoma (LUAD), are commonly driven by specific genetic alterations in oncogenes leading to abnormal proteins that can be targeted (2). The discovery of actionable mutations in NSCLC has changed the treatment paradigm from cytotoxic chemotherapy to molecular-targeted therapy.

Epidermal growth factor receptor (*EGFR*) is the most common driver oncogene in NSCLC especially in Asian patients (3, 4). Both exon 19 deletions (19 del) and the point mutation L858R at exon 21 are common mutations, accounting for more than 85% of *EGFR*-mutant NSCLC, which predict sensitivity to EGFR-tyrosine kinase inhibitors (TKIs) (5). Uncommon mutations such as *EGFR* T790M mutation and exon 20 insertions have been documented to predict resistance to EGFR-TKI. However, whether patients harboring other uncommon mutations accounting for about 10% of all *EGFR* mutations obtain clinical benefit from EGFR-TKI is seldom investigated because in the majority of clinical trials investigating the efficacy of EGFR-TKIs, only patients with sensitizing *EGFR* mutations, 19 del, and L858R, are included (6, 7). Here, we presented a patient with metastatic NSCLC harboring *EGFR*-kinase domain duplication (KDD) who derived durable response to first-line treatment of second-generation EGFR-TKI afatinib with a progression-free survival (PFS) of 12 months and counting.

## Case Presentation

**Figure 1:**
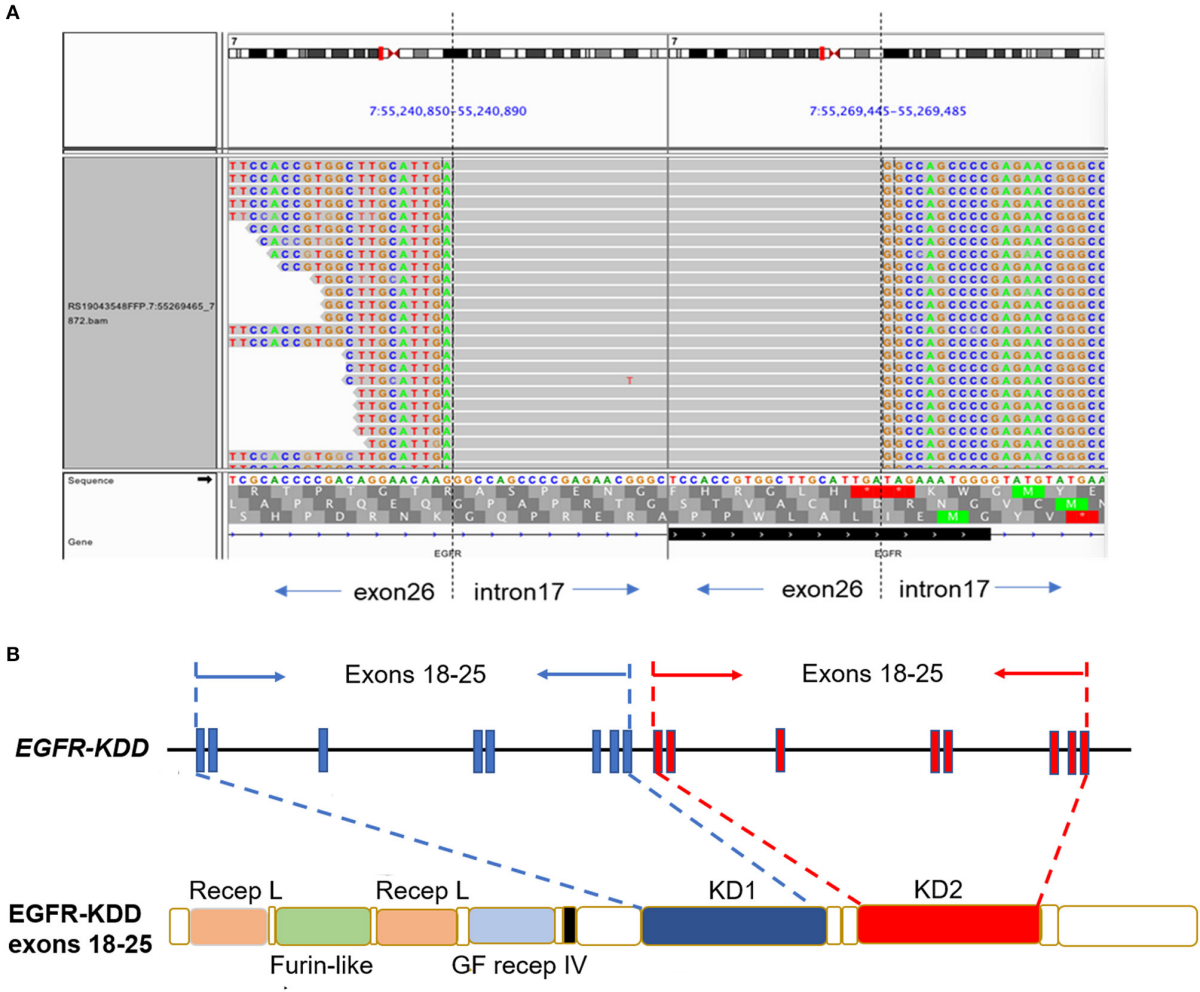
The LUAD patient harboring canonical *EGFR-*KDD involving exons 18–25. **(A)** Visualization of canonical *EGFR-*KDD using the Integrative Genomics Viewer (IGV) browser. The dashed lines indicate the genomic breakpoints. **(B)** The genetic and protein domain structures of *EGFR-*KDD. *EGFR*/EGFR, epidermal growth factor receptor; KDD, kinase domain duplication; LUAD, lung adenocarcinoma; Recep L, Receptor L domain; Furin-like, Furin-like cysteine rich region; GF recep IV, Growth factor receptor domain IV; KD, tyrosine kinase domain.

**Figure 2:**
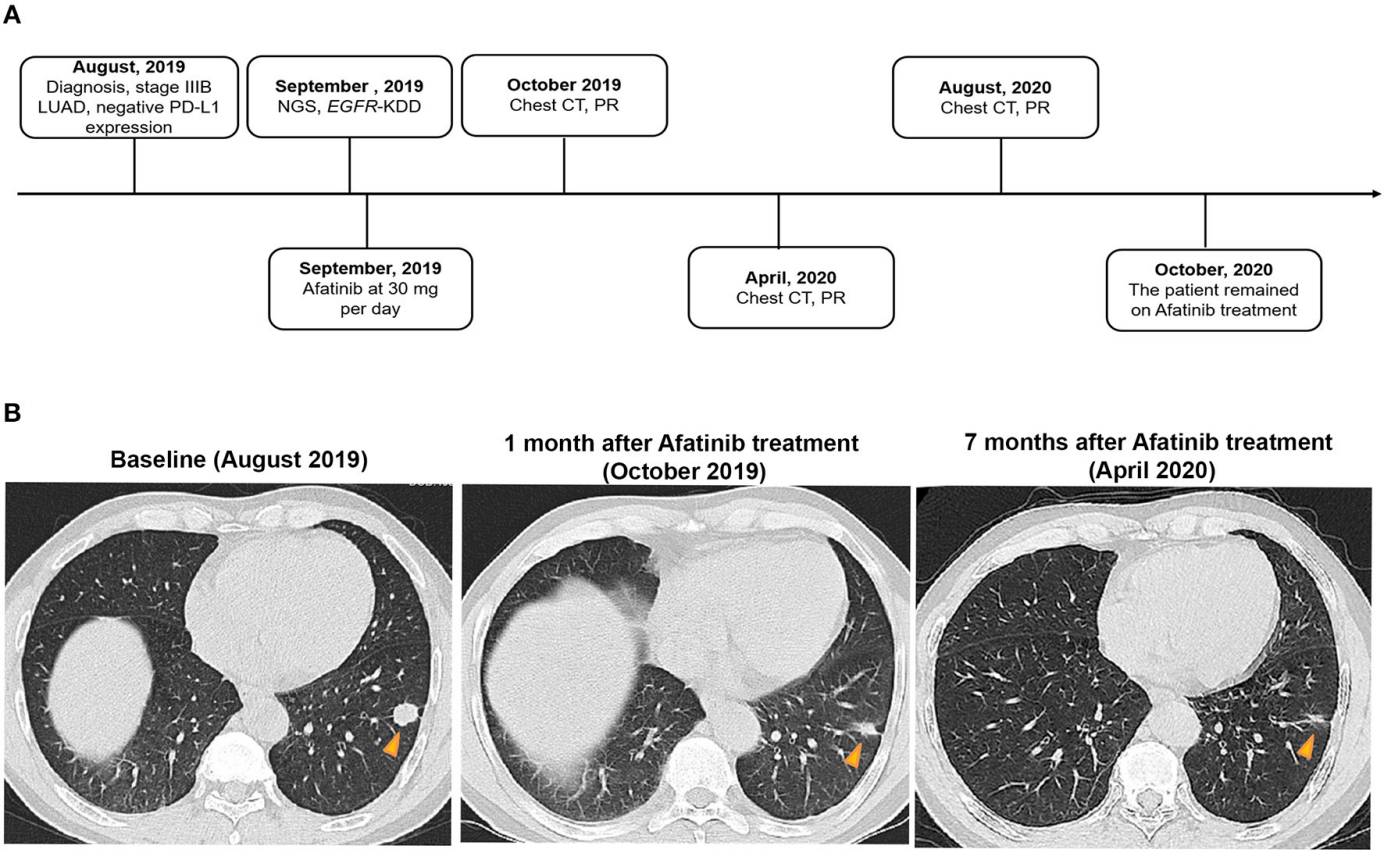
A summary of the treatment procedure of the patient. **(A)** The entire treatment procedure. LUAD, lung adenocarcinoma; PR, partial response; NGS, next-generation sequencing; *EGFR*, human epidermal growth factor receptor 2; KDD, kinase domain duplication; CT, computed tomography; PD-L1, programmed death ligand-1. **(B)** Chest CT imaging of the LUAD patient prior to afatinib treatment, 1 month after the treatment, and 7 months after the treatment. The yellow arrows indicate the lesions. LUAD, lung adenocarcinoma; CT, computed tomography.

A 61-year-old man with a smoking index of 600 (15 cigarettes/day for 40 years) presented with a cough for 2 weeks. He had no family history of cancer. Chest CT scans revealed a mass located in the lower lobe of the left lung, and a CT-guided percutaneous lung biopsy revealed LUAD. In addition, the ultrasound revealed the enlargement of the left and right supraclavicular lymph nodes (SCLNs). Subsequently, an ultrasound-guided percutaneous biopsy of the right SCLNs was performed and the patient was diagnosed with T1N3M0 (IIIB) LUAD in August 2019. The patient had an Eastern Cooperative Oncology Group performance status (ECOG PS) of 1. Immunohistochemistry testing (IHC 22C3) for programmed death ligand-1 (PD-L1) was performed on the primary tumor biopsy. IHC analyses revealed the patient was negative for PD-L1 expression with membranous expression of PD-L1 on <1% of tumor cells. Capture-based targeted sequencing (Burning Rock Biotech, Guangzhou, China) was performed on the primary tumor sample, which revealed canonical *EGFR*-KDD involving exons 18–25 (Figure 1) and negative for well-known actionable alterations occurring in *EGFR*. He refused to receive chemotherapy and was administered with afatinib (30 mg per day, orally) in September 2019. Figure 2A illustrated the treatment procedure of the patient. The patient achieved partial response (PR) with significant shrinkage of the tumor, from a diameter of 17 mm to 11 mm 1 month after afatinib treatment and it further reduced to 6 mm at 7 months (Figure 2B). The ECOG PS of the patient decreased to 0 after 3 months of afatinib treatment. In August 2020, chest CT still showed a PR after 11 months of afatinib treatment. Afatinib was well-tolerated, with grade 1 rash that did not require medical treatment for control and grade 2 diarrhea (3–4 times per day) that disappeared after symptomatic treatment for 2 months. There were no treatment-related adverse events leading to discontinuation. As of the submission of this manuscript, the patient still remained on the treatment, with a PFS of 12 months and counting. The patient was satisfied with the effect of EGFR-TKI afatinib treatment.

We also reviewed the previously reported six cases harboring *EGFR-*KDD, of which three cases showed PR to first- or second-generation EGFR-TKI treatment. The clinical characteristics and outcomes of patients with NSCLC harboring *EGFR*-KDD before TKI treatment in our and previous studies are summarized in Table 1.

**Table 1:** Clinical characteristics and outcomes of patients with NSCLC harboring *EGFR*-KDD in our and previous studies.

**Pt No**.	**Publication**	**Year of publication**	**Age**	**Gender/** **Ethnicity**	**Diagnosis/** **Stage**	**EGFR-TKI** **Treatment/line No**.	**Concurrent mutations**	**Response to TKI**	**PFS**
1	Gallant et al. (8)	2015	33	Male/American	LUAD/IV	Afatinib/2nd line	None	PR	7 cycles of therapy
2	Zhu et al. (9)	2018	63	Female/Chinese	LUAD/IV	Icotinib/1st line	NA	SD	11 months, NR
3	Wang et al. (10)	2019	61	Male/Chinese	LUAD/IV	Erlotinib/2nd line Osimertinib 3rd line	*TP53* R280G	PD PD	2 months 2 months
4	Wang et al. (10)	2019	63	Male/Chinese	LUAD/IV	Gefitinib/1st line Afatinib/2nd line Osimertinib/3rd line	*ERBB2* amp	PR PD PR	5 months 2 months 4 months, NR
5	Wang et al. (10)	2019	67	Male/Chinese	LUAD/IV	Icotinib/2nd line	*TP53* Y220C *PIK3CA* E81G	PR	4 months, NR
6	Chen et al. (11)	2020	59	Male/Chinese	LUAD/IV	Afatinib/1st line	*TP53* R282W *CTNNB1* S37Y	SD	10 months, NR
7	Our study		61	Male/Chinese	LUAD/IIIB	Afatinib/1st line	None	PR	12 months, NR

*EGFR, epidermal growth factor receptor; KDD, kinase domain duplication; NSCLC, non-small cell lung cancer; PD, progressive disease; PR, partial response; SD, stable disease; NA, not available; NR, not reached; Pt, patient; No., number; LUAD, lung adenocarcinoma; PFS, progression-free survival; TKI, tyrosine kinase inhibitor*.

## Discussion

This study presents the clinical evidence of a patient with *EGFR*-KDD driven metastatic LUAD benefiting from afatinib as the first-line treatment with a PFS of 12 months and counting. To the best of our knowledge, this is the longest PFS among all reported studies.

Epidermal growth factor receptor- kinase domain duplication as the rare alteration is identified only in 0.12% (12/10,759) of all NSCLCs and 0.24% of all *EGFR*-mutant patients in East Asian population (10). KDD is a special type of large genomic rearrangements occurring in the kinase domain of protein kinase genes, which results in a novel mechanism for protein kinase activation in tumor cells. *EGFR*-KDD as the most well-studied KDD often results from in-frame tandem duplication of *EGFR* exon 18–25, causing subsequent constitutive activation of EGFR signaling (8). Preclinical data demonstrate that *EGFR*-KDD confers constitutive activity to the EGFR tyrosine kinase and sensitivity to EGFR-TKIs including erlotinib, afatinib, and osimertinib (8). Several instances of clinical evidence have revealed the efficacies of EGFR-TKIs in advanced LUAD but yielded variable antitumor responses (8–11). The first case of *EGFR*-KDD in LUAD is reported by Gallant et al. where a 33-year-old male smoker diagnosed with stage IV LUAD did not carry any common *EGFR* mutations but achieved PR after being treated with afatinib (8). Stable disease (SD) response to afatinib was observed in a 59-year-old patient with LUAD (11). Furthermore, studies have also documented that patients with *EGFR*-KDD-positive LUAD showed response to first-generation EGFR-TKI icotinib and third-generation EGFR-TKI osimertinib (9, 10). Baik et al. reported a female patient with bronchoalveolar carcinoma achieved a durable response to gefitinib and erlotinib, and *EGFR*-KDD was detected in the advanced stage (12), we cannot determine whether this *EGFR*-KDD is a primary or a secondary alteration after TKI treatment. However, in contrast, an *EGFR*-KDD-positive LUAD patient refractory to EGFR-TKIs has also been reported (10), a 61-year-old male patient harboring *EGFR*-KDD of exon 18–25 concurrent with *TP53* R280G mutation progressed shortly after undergoing therapies of erlotinib and osimertinib for only 2 months. Among seven cases harboring *EGFR-*KDD (including the current case), four cases achieved PR response to first- or second-TKIs treatment, and reached an objective response rate (ORR) of 57%. More evidence or clinical trials are needed to evaluate the efficacy of EGFR-TKIs in patients with LUAD harboring *EGFR*-KDD.

Previous studies have revealed that afatinib-related adverse events occur in more than 97% of patients with NSCLC (6, 7). Diarrhea (88.3%) and rash (80.8%) are the most common adverse events (7). In the present study, grade 1 rash and grade 2 diarrhea were reported by the patient. This patient had good adherence to the afatinib treatment. The rash did not require medical treatment for control. The diarrhea disappeared after symptomatic treatment for 2 months. Afatinib was well-tolerated and effective in the patient with advanced LUAD harboring *EGFR*-KDD.

A few limitations are associated with our study. Due to the nature of research, only one patient was incorporated into the work. Large cohort studies or clinical trials should be launched to verify the efficacy and safety of afatinib as the first-line treatment for patients with advanced LUAD harboring *EGFR*-KDD.

Here, we reported a patient who was *EGFR*-KDD-positive and showed durable response to EGFR-TKI therapy, thereby confirming *EGFR*-KDD is an oncogenic driver and a therapeutic target. Our work provided clinical evidence to administer EGFR-TKI in advanced NSCLC patients harboring *EGFR*-KDD and paving the way for its potential clinical utilization.

## Data Availability Statement

The raw data supporting the conclusions of this article will be made available by the authors, without undue reservation.

## Ethics Statement

The studies involving human participants were reviewed and approved by the Ethic Committee of the Second Affiliated Hospital of Zhejiang University School of Medicine. The patients/participants provided their written informed consent to participate in this study. Written informed consent was obtained from the individual(s) for the publication of any potentially identifiable images or data included in this article.

## Author Contributions

PW designed the study and wrote the manuscript. LZ and ZW involved in diagnosing, treating, providing follow-up for the patient, and collecting data for this report. HD and SC conducted the genetic test and analysis. All authors read and approved the final version of the manuscript for submission.

## Conflict of Interest

HD and SC were employed by Burning Rock Biotech. The remaining authors declare that the research was conducted in the absence of any commercial or financial relationships that could be construed as a potential conflict of interest.
